# Inactivation of model viruses and bacteria in human fresh frozen plasma using riboflavin and long wave ultraviolet rays

**Published:** 2017-02

**Authors:** Ameneh Elikaei, Seyed Masoud Hosseini, Zohreh Sharifi

**Affiliations:** 1 Department of Microbiology, Faculty of Biological Sciences, Alzahra University, Tehran, Iran; 2 Department of Microbiology, Faculty of Biological Sciences & Technologies, Shahid Beheshti University, Tehran, Iran; 3 Blood Transfusion Research Center, High Institute for Research and Education in Transfusion Medicine, Tehran, Iran

**Keywords:** Viruses, Bacteria, Fresh frozen plasma, Riboflavin, Ultraviolet rays, Inactivation

## Abstract

**Background and Objectives:**

Pathogen reduction technologies are among methods to eliminate transfusion transmitted infections. Mirasol method using riboflavin in combination with ultraviolet rays is one of them. The aims of this study were to investigate the effectiveness of Mirasol method to inactivate some model pathogens as well as examination of the sensitivity of plasma proteins after treatment.

**Materials and Methods:**

Riboflavin in 50μM concentration and ultraviolet (365 nm) in three different energy doses (3.6, 7.2, and 10.8 j/cm^2^) were employed to inactivate model pathogens. Four standard viruses were used in this study including *Vesicular Stomatitis Virus* (VSV), *Herpes Simplex Virus1* (HSV-1), *Bovine Viral Diarrhea Virus* (BVDV) and *Polio Virus*. 50% Tissue Culture Infectious Dose (TCID_50_) and Reed–Muench Methods were used to estimate viruses’ titers. *E. coli* and *Staphylococcus aureus* were used as bacterial models. Four plasma proteins including factor V, VIII, fibrinogen and antithromin were used to determine their sensitivity to pathogen inactivation treatment.

**Results:**

The most pathogen reduction titre was determined for 15 minutes irradiation period equal to 10.8 J/cm^2^ that is corresponding to Log 6.10 for BVDV, Log 6.09 for HSV-1, Log 6.62 for VSV and Log 3.36 for Polio. Bacterial reduction titer was Log 6.94 for *E. coli* and Log 7.00 for *S. aureus*. Indicator proteins for plasma activity were determined to be 75% for factor V, 88% for factor VIII, 52% for fibrinogen and 94% for antithrombin.

**Conclusion:**

Results showed that the employed method can inactivate most of the pathogens in fresh frozen plasma. The acceptable activities of selected plasma proteins remained after treatment.

## INTRODUCTION

Viral and bacterial contamination cause many problems in blood components transfusion. In spite of serological and molecular screening methods and also proper blood donor selection, still some risks for blood component transfusion remained (1). To avoid window period misdiagnosis of viruses such as *Human Immunodeficiency Virus* (HIV) and *Hepatitis C Virus* (HCV) and emerging or re-emerging viruses such as *West Nile Virus or Chikungunya Virus* which there is no method to detect them (2, 3).

Several pathogen reduction technologies (PRT), have been developed to prevent transfusion transmitted infections. Some methods using photosensitisers as a key character, which absorb light energy and, in the ensuing electronically excited species, undergo redox reaction with surrounding molecules. Most current methods target nucleic acids and lead to pathogen inactivation (4).

Riboflavin in combination with long wave Ultra Violet (UV) is one of the effective PRTs. Riboflavin as Vitamin B2, is an essential nutrient in humans and it is generally recognized as Safe (GRS) (5). The safety of it has been demonstrated for oral, subcutaneous, intra-peritoneal and intravenous routes of administration (6–8). Mirasol system using riboflavin in combination with UV inactivates pathogens in blood components. Treatment of blood components employing this method has reduced significantly the risk of pathogen transmission (2, 5, 9–11). Its effectiveness for some pathogens has not been investigated. In this study, we used it as a new source for UV and investigate its effectiveness on four model viruses and two model bacterial pathogens. The impacts of this treatment on some proteins in Fresh Frozen Plasma (FFP) were also investigated.

## MATERIALS AND METHODS

### Viruses and cell lines.

Four model viruses, *Bovine Viral Diarrhea Virus* (BVDV), *Herpes Simplex Virus 1* (HSV-I), *Vesicular Stomatitis Virus* (VSV) and *Poliomyelitis Virus* (Polio), to represent enveloped, non-enveloped and both RNA and DNA V Viruses were selected for this study. All four strains obtained from virus bank of Iranian Blood Transfusion Organization (IBTO) (12).

Madin-Darby Bovine Kidney (MDBK), African Green Monkey Kidney (Vero), and Cervix Carcinoma (HeLa) cell lines were used to propagate and quantify model viruses. All cell lines were obtained from National Cell Bank of Iran (NCBI), Pasteur Institute of Iran. The cells were grown in monolayers in Dulbecco Modified Eagle Medium (DMEM) (Gibco, UK) with 5% Fetal Bovine Serum (FBS) (Gibco) and 1% Pen-Strep (Sigma) at 37°C in a humid atmosphere of 5% CO_2_.

### Virus propagation and quantification.

50% Tissue Culture Infectious Dose (TCID_50_), were used to quantify viruses. Model viruses propagated in mono-layer cell lines and their infectivity titer were determined by Reed and Muench method employing 10 fold dilution series four replicate culture per dilution (13). Each virus titre should be reached at least 10^6^ according to WHO guidelines for PRT (Pathogen Reduction Technologies) (14). Then virus stock has been frozen at −70°C until used.

### Bacteria.

With respect to the most common bacterial contamination in blood transfusion two Gram-positive and Gram-negative bacteria were selected for this study: *E. coli* (ATCC:25922) and *S. aureus* (ATCC: 25923). They cultured in nutrient agar for 24 hours at 37°C. Then a suspension of each organism was prepared in sterile phosphate buffer saline (PBS) (pH=7.4) to reach 10^6^ CFU/ml.

### Fresh frozen plasma (FFP).

FFP samples have been obtained from IBTO. They were negative for HIV (Human Immunodeficiency Virus), HCV (Hepatitis C Virus) and HBV (Hepatitis B Virus) and stored at −20°C until used. All of them were O positive.

### Plasma proteins.

To determine the quality of FFP before and after riboflavin and UV treatment, 4 plasma proteins including factor V, factor VIII, antithrombin and fibrinogen were selected as indicators to examine their concentration before and after treatment.

### Riboflavin.

Riboflavin was obtained from Darou Pakhsh Holding Co and stored at 25°C in dark before use. After that it was suspended in PBS and used in 50 μM final concentration in FFP samples.

### Irradiation source.

The source of irradiation was a UV Irradiator from HAMAMATSU (lighting lure series LC5 model). It could irradiate UV in 200–400 nm and its optimum irradiation was in 365nm. The energy dose in samples depends on their distance from the source as 12cm distance designed to reach optimum energy and also 3 different exposure times of 5, 10 and 15 minutes were employed to obtain different energy dose to identify optimum dose for inactivation of pathogens. The final calculated energy dose for each exposure time were 3.6, 7.2, and 10.8 j/cm^2^ respectively.

### Pathogen inactivation procedure.

Nine milliliter of FFP samples were added to 9cm petri-dishes and 1ml of bacteria and virus stocks were suspended in FFP to reach at least 10^6^ TCID_50_ for virus and 10^6^ CFU for bacteria per ml. Then riboflavin in a final concentration of 50μM was added to each sample (5). The irradiation procedure was done in microbiological safety cabinet level II to prevent cross contamination. Virus and bacteria titers before and after irradiation was examined using TCID_50_ and Reed and Muench method for viruses and serial 10- fold dilutions in nutrient agar for bacteria.

### Controls.

Negative controls were designed to be FFP without any pathogen spiking and also relative cell lines and bacterial media without pathogen spiked them. Positive controls were FFP contained pathogens without treatments.

### Statistical methods.

All experiments repeated 3 times. SPSS (version 20.Inc) was used for data analysis.

## RESULTS

### Viruses.

The original titer of BVDV, HSV, VSV and Polio Viruses were 6.54, 6.82, 7.07 and 6.66 respectively. Using riboflavin in 50μM final concentration in associated with UV in 365nm and in three different times reduced BVDV log in FFP samples to 6.10 (Table 1).

**Table 1.** Inactivation of 4 model viruses (BVDV, HSV, VSV and Polio) in FFP using 50μM of riboflavin in combination with UV irradiation exposure time.

**Times of irradiation**	**Log reduction (Mean± SD)**			

	BVDV	HSV	VSV	Polio
5 min	3.07±0.24	3.52±0.343	4.6±0.25	1.06±0.10
10 min	5.02±03	4.44±0.28	5.46±0.12	2.11± 0.05
15 min	6.10± 0.09	6.09±0.14	6.62±0.09	3.36±0.17

For HSV-1 best log reduction was 6.09 for 15 minutes irradiation, and VSV with the same exposure times showed a 6.62 log reduction. Polio virus was the most resistance virus that showed only a 3.36 log reduction after 15 minutes irradiation in presence of the riboflavin. The results were compared to the controls.

### Bacteria.

The original titer for *E. coli* was 10^7.7^ CFU/ml and for *S. aureus* was 10^7.0^ CFU/ ml. *E. coli* showed Log 6.94 CFU/ml reduction after 15 minutes treatment and *S. aureus* howed more sensitivity to riboflavin and UV treatment that log 7 CFU/ml reduction has been shown after 10 minutes (Table 2).

**Table 2.** Inactivation of *E. coli* and *S. aureus* in FFP using 50μM concentration of riboflavin and UV irradiation.

**Times of irradiation**	**Log reduction for** ***E. coli*****, CFU/ml Mean± SD**	**Log reduction for** ***S. aureus*****, CFU/ml**
5 min	2.7±0.01	3.64±0.11
10 min	5.67±0.06	7.00±0.09
15 min	6.94±0.12	7.00±0.15

### Plasma proteins.

The results showed selected plasma proteins remained in normal range after riboflavin in combination with UV treatment except fibrinogen which was the most sensitive factor to this method. Antithrombin was the most resistance one. Table 3 shows the average activity of the proteins before and after treatment.

**Table 3.** Impact of 50 μM riboflavin concentration in association with 10 minutes ultraviolet treatment on FFP proteins.

**Protein**	**Average of activity before irradiation**	**Average of activity after 10 m irradiation**	**Normal range**	**Percent of remaining activity after treatment**
Factor V %	110	82.3	60–130	75%
Factor VIII %	89	78.5	60–150	88%
Fibrinogen mg/ml	323	170.5	200–400	52%
Antithrombin %	114	108	80–120	94%

## DISCUSSION

Blood-borne pathogens removal or reduction to the acceptable level always considered as a critical point in blood transfusion process. Although the efficiency of present methods, including screening blood donor and testing blood samples are useful to improve blood component quality, but still remain some risks, especially because of emerging or re-emerging viruses and critical window period for detection of some viruses (1, 5, 15). So, new PRTs have been developed to reduce more blood-borne pathogens transfusion risk. In this study, using riboflavin in combination with UV emission at the certain wave length as a PRT method was examined. The effectiveness of this method was examined using four viruses model viruses and two bacteria model. In the Mirasol system 50μM concentration of riboflavin and UV with 6.24 J/ml energy dose used to inactivate pathogens. Three different energy doses of 3.6, 7.2, and 10.8 j/cm^2^ were used. Marschner and Goodrich showed VSV titer reduced 6.3 Log using the Mirasol method in comparison with current study although they use Sindbis Virus as a model for HCV that showed a 3.2 log reduction, in this study BVDV model virus for HCV recommended by WHO guidelines was used and showed a 6.10 Log reduction using 10.8 j/cm^2^ energy dose (5, 16).

In current study Poliovirus employed as a model virus for non- enveloped that showed more resistant to this PRT method, in this way, other study shows Human B19 Parvo virus 5 Log reduction after using Mirasol method. There are no published data available on HSV and VSV reporting the sensitivity to riboflavin in combination with UV blood-borne pathogens removal or reduction to acceptance level (5, 16).

The bacterial selection in this study was based on their high contamination rate in blood transfusion. Previous studies showed *Staphylococcus epidermis*, *S. aureus* and *E. coli* sensitivity to Mirasol method. In current study the same results for *E. coli* and *S. aureus*, was observed. However, *E. coli* showed high sensitivity to treatment, as more than 6 Log reduction after 15 minutes was observed (5, 10, 11).

Marsh and et al. showed antitrombin can save 90% of its activity after using the Mirasol method and is the most resistant protein and fibrinogen lost 30% of its activity and is the most sensitive one. The current results were near to it and all plasma factors remained in normal range after pathogen inactivation (5, 17).

The results showed the PRT treatment could inactivate most pathogens including both bacterial and viral contamination at the much closed level to WHO guideline, and is a safe and acceptable method for pathogen reduction in blood transfusion centers. A 10.8 J.cm^2^ is the most effective energy dose, although, 7.2 J/cm^2^ still is remaining as an alternative to save more plasma proteins’ activity.
